# A Novel Anti-Spoofing Solution for Iris Recognition Toward Cosmetic Contact Lens Attack Using Spectral ICA Analysis

**DOI:** 10.3390/s18030795

**Published:** 2018-03-06

**Authors:** Sheng-Hsun Hsieh, Yung-Hui Li, Wei Wang, Chung-Hao Tien

**Affiliations:** 1 Department of Photonics, National Chiao Tung University, 1001 University Road, Hsinchu 30010, Taiwan; jack10313.eo00g@nctu.edu.tw (S.-H.H.); wwang107@gmail.com (W.W.); 2 Department of Computer Science & Information Engineering, National Central University, 300 Zhongda Road, Zhongli District, Taoyuan 32001, Taiwan; yunghui@csie.ncu.edu.tw

**Keywords:** spectral imaging, independent component analysis, iris recognition

## Abstract

In this study, we maneuvered a dual-band spectral imaging system to capture an iridal image from a cosmetic-contact-lens-wearing subject. By using the independent component analysis to separate individual spectral primitives, we successfully distinguished the natural iris texture from the cosmetic contact lens (CCL) pattern, and restored the genuine iris patterns from the CCL-polluted image. Based on a database containing 200 test image pairs from 20 CCL-wearing subjects as the proof of concept, the recognition accuracy (False Rejection Rate: FRR) was improved from FRR = 10.52% to FRR = 0.57% with the proposed ICA anti-spoofing scheme.

## 1. Introduction

Iris recognition has drawn much attention because of its well-established framework, extremely high accuracy, and computational efficiency [1,2,3]. Recently it is becoming even more popular and has started to be deployed for national-scale applications [4,5]. Like other biometric identification, iris recognition is subject to attempted forgery challenges. Intentionally or not, when people wear cosmetic contact lenses (CCLs), whose circular pigment may fully or partially obscure the natural iris texture, the recognition performance will be degraded accordingly.

Many efforts have been made to develop anti-spoofing techniques for iris recognition. Generally speaking, spoofing techniques can be divided into (1) using a completely fake or printed eye to fool the system; and (2) modifying the texture of a living iris using augmented material, which, for most cases, in iris recognition involves wearing CCLs. Therefore, the anti-spoofing technique can also be divided as: (1) liveness detection: to classify whether the eye under test is alive; (2) CCL detection: to classify whether the eye under test is wearing CCL. For the case of liveness detection, if the eye under test is classified as a fake eye, the iris recognition process simply stops and the system simply rejects the current subject and waits for the next. For the case of CCL detection, when the system detects that the subject wears a CCL, it will most likely require the user’s cooperation to take CCL off and then performs recognition again. To our best understanding, currently there is no anti-spoofing technique which claims to be able to perform iris recognition directly on the CCL-wearing subjects. In this paper, we have therefore attempted to develop a hardware-software hybrid iris recognition system which deals with CCL-based spoofing. Based on the assumption that the iris texture and CCL pattern are statistically independent in the spectral domain, the proposed system, which combines a dual-band camera (DBC) system with a source separation technique (Independent Component Analysis, ICA), has the capability of separating the spectral component of the natural iris region from the CCLs’ textures, followed by removing the CCL texture from the mixed images. As a consequence, there is no need to ask the subject to take off CCL and the system is capable of performing iris recognition directly based on the mixed images. Such a technique is not detection-based, but rather recognition-based, which we consider as the major contribution of our work. 

The remainder of this paper is organized as follows: in Section 2, we present a literature review to give an overview of anti-spoofing technique for iris recognition. In Section 3, we describe our dual-band camera (DBC) system with the corresponding ICA model. In order to discount the incidental variations caused by CCLs from the natural iris images, we optimize the mixing matrix to ensure that two spectral images have the largest independency in statistics. In Section 4, we introduce the algorithm to solve the ambiguity issues of ICA, thus determine the mask pattern to remove the influence from the CCLs. Some subtle considerations to examine the validity of ICA methodology on iris recognition are made. Preliminary results in test are presented in Section 5 prior to the summary and conclusion in Section 6.

## 2. Iridal Texture Characteristics and Anti-Spoofing Technique Overview

### 2.1. Iridal Texture Characteristics

**Figure 1:**
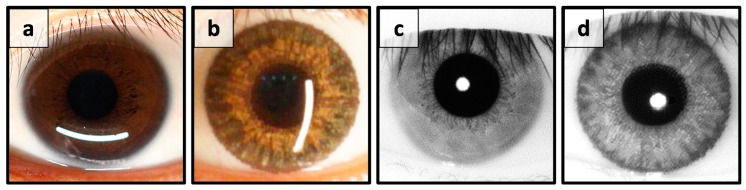
Images of same eye with and without CCL; (**a**,**b**) captured by color sensor and (**c**,**d**) monochromatic sensor; (**a**,**c**) naked eye and (**b**,**d**) with CCL.

The iris is the flat and circular membrane behind the cornea of an eye. The main function of the iris is to control the amount of luminous flux through the pupil. The pupil varies in size from 3 to 8 mm by manipulation of two pupillary muscles, namely the dilator and sphincter pupillae [6]. Generally, the iris of an adult spans approximately 12 mm in diameter and 0.5 mm in thickness. The iris spectral reflection, also called albedo, is dependent on the composition of iridal pigments. Those of most East Asians appear dark brown, where the albedo reflection in the visible spectrum is pretty low (about 0.05–0.2) but rather high (about 0.4–0.5) under near infrared (NIR) illumination [7,8,9]. That is the main reason why the illumination used for most iris recognition techniques falls in the NIR spectrum. As shown in Figure 1, the natural iris texture from an East Asian subject is barely observable under visible light (Figure 1a) but is more obvious under NIR (Figure 1c). When the same subject puts on a CCL, compared with the natural iris, the pigments of the commercial CCL are conspicuous under both visible and NIR illumination (Figure 1b,d). It is noted that the CCL pattern is partially overlaid on top of the natural iris texture, thus the mixed images would substantially deteriorate the accuracy of any iris recognition system under both visible and NIR illumination conditions.

### 2.2. Iris Anti-Spoofing Technique Overview

Generally speaking, the anti-spoofing techniques for iris biometrics can be roughly divided into two categories: liveness detection and CCL detection

#### 2.2.1. Liveness Detection

Liveness detection is a preventative approach for sensor-level attacks in biometric systems in which malicious users construct false replicas of legitimate biological characteristics, applying them directly to the sensors and declaring their corresponding identities. The major goal for the technique of iris liveness detection is to determine whether the current eye under test is a true eye or not. According to [10], the technique of liveness detection and tamper detection methods are considered as presentation attack detection (PAD) methods which are used to detect spoofing attempts on the biometric system [11,12,13,14,15]. PAD technique has becoming more and more important in recent years as biometric authentication mechanism has been applied more on consumer electronic devices and access control system for exclusive areas. Generally speaking, liveness detection technique can be distinguished into hardware-based and software-based systems.

##### Hardware-Based Systems

Hardware-based systems make use of optoelectronic sensors that are additional to the biometric systems to test whether the iris under inspection is alive or not [16,17,18]. The liveness detection is mainly based on whether important physiological characteristics of eyeballs can be positively detected, for example, tissues or blood vessels in the sclera.

##### Software-Based Systems

Software-based systems do not require auxiliary electrical or optical sensors to attach to the biometric system [19,20,21,22,23,24,25,26]. It basically analyzes clues exhibited in the images that are captured by the biometric system and uses algorithmic approach to perform binary classification (alive or not alive). The detection process can be further divided into passive detection and active detection. Passive detection is to observe whether the eyeballs exhibit the natural response, such as eye hippus or natural oscillation of the pupil sizes. One of the early work stated in [27] suggested that “red-eye” effect can be utilized as an effective clue to detect fake eyes. Another feasible solution is by analyzing the frequency spectrum. As also mentioned in [27], printed iris images have intrinsic artifacts that can be detected using 2D Fourier transform. The active detection is to observe whether the eyeballs react as the way they should when external stimuli is applied, for example, asking the users to blink or look in a specific direction.

#### 2.2.2. Cosmetic Contact Lenses (CCL) Detection

Different from other biometric modalities, for iris recognition, malicious users can try to wear CCL to forge iris textures in order to fool the system. Due to the transparency characteristics of CCL, the captured images of the eye wearing CCL present patterns from both CCL and iris, resulting in a mixed image pattern. In this way, the eye that wears CCL is still alive, but the pattern of the iris may be changed or partially modified. Therefore, we consider this case different than the case of liveness detection. We categorize such cases as “CCL detection”. For the case of CCL detection, in literature, researchers addressed more on how to detect whether the subjects were wearing CCL. Generally, the counter measure can be divided into two approaches. One is based on the physiology-based detection [28,29,30,31,32,33]. The key point of this scenario lies in the dissimilarity of appearance primitives between natural iris and CCLs due to their physiological features, such as pupil dynamics under illumination modulation (temporal domain), three dimensional (3D) shape or glare reflection position (spatial domain), or spectral reflection (spectral domain). The other approach comes with the learning-based methodology through the pure software work [33,34,35,36,37,38,39,40,41,42,43,44]. Feature extraction with a texture-based classifier was performed to automatically detect if the subject is wearing a CCL.

##### Physiology-Based Detection

For the physiology-based detection, Lee et al. [30] employed a NIR illuminator to inspect the spots of different Purkinje images according to Gullstrand’s eye model. The Purkinje images contain specular reflection points occurred at various layer boundaries: anterior cornea (first), posterior cornea (second), anterior lens (third), and posterior lens (fourth). Each Purkinje image had its own specific position for detection in a searching window. Likewise, Park et al. [31] detected the pupillary boundary change through the flash of illumination. The study evaluated the hippus movement (i.e., the ratio of dilation to contraction of the pupil) and successfully circumvented the false iris of printed or artificial eyes. However, since the circular band from the CCLs might fully occlude the pupillary boundary, the feasibility of this method against the CCL-based forgery has not proved yet. Lee et al. [32] proposed to measure the variation of the albedo ratio between the iris and the sclera under different illumination wavelengths. This method was proved successful to tackle printed and artificial forgery with 750 nm (visible) and 850 nm (NIR)-centered illumination. However, this method was unlikely to be applied in a universal approach for various contact lens colors and races. Hughes et al. [33] used the stereo imaging to distinguish the 3D shape between the natural iris and CCL. The natural iris region is relatively planar. When users wore a CCL, the change of perceived curvature was used as a depth cue to decide if a CCL was present. This work needed larger disparity for depth estimation, thereby unlikely to work in a long-range recognition system. Also, the capture volume in object space where the eye can be positioned for successful image was extremely small, thus requiring the user cooperation in operation.

##### Texture-Based Classifier

Daugman [34] pioneered a countermeasure against dot-matrix CCL forgery by using a 2D Fourier transform. The 2D power spectrum of a CCL gave rise to four strong responses at high frequencies. This method was validated merely for the CCLs those were utilized in the limited training set. Furthermore, the detection of spatial frequency from the dot matrix pattern was limited when it exceeded the Nyquist frequency of the sensor, or when images were moving blurred, out of focus, or otherwise imperfect imagery condition. He et al. [35] devised a statistical texture analysis that employed gray level co-occurrence matrices (GLCMs) [36] to extract the textural features, then classified them through the popular support vector machine (SVM). The GLCMs recorded pairs of neighboring pixels with specific values. The features were extracted according to the contrast and angular second moment from the GLCMs; the mean and standard deviation were derived from the normalized iris image. Four distinctive features were classified using SVM. This study provided a robust algorithm for automatically detecting CCLs in iris images. The major problem lies in the requirement of heavy computation and prior knowledge about CCL sample, therefore hinders its practical use in real time. Wei et al. [37] characterized CCLs by using the texton model. The authors used the Gabor filter to extract the features from a mixed iris image. The Gabor filter has favorable properties in both orientation and spatial frequency. A total of 40 even Gabor filters produced a feature vector with 40 dimensions. By clustering these feature vectors with *K* means (*K* was set to 64), the mixed iris image could be represented by an iris texton histogram. A classifier that exploited the distinctness of the natural iris texture and CCL texton patterns was thus devised. Like the case in unique filter and SVM, heavy computation is challenging for real-time operation. To speed up the computation, He et al. [23] proposed using a local binary pattern (LBP) to extract features in the iris. The LBP was an effective texture descriptor for creating an image histogram based on the image texture. The authors employed a window for observing a local part of the image. For each pixel in the window, compared with each of its eight neighbors, the value was set as 1 if the center pixel value was greater than the neighbor’s value, and otherwise was set as 0. This operation produced an eight-digit binary number, which normally was presented as a decimal. After each part of the region of interest in the image was processed, its histogram could be computed and employed in the form of a vector feature. Using these features, on the basis of GLCMs or LBPs, and with one or several trained classifiers (e.g., AdaBoost), this method could distinguish whether the iris image contained a CCL [38]. More recent works based on learning algorithm are underway [39,40,41,42,43]. However, a compromise must be reached between the countermeasure capability and the computational complexity.

### 2.3. Summary of Iris Antispoofing Technologies

**Figure 2:**
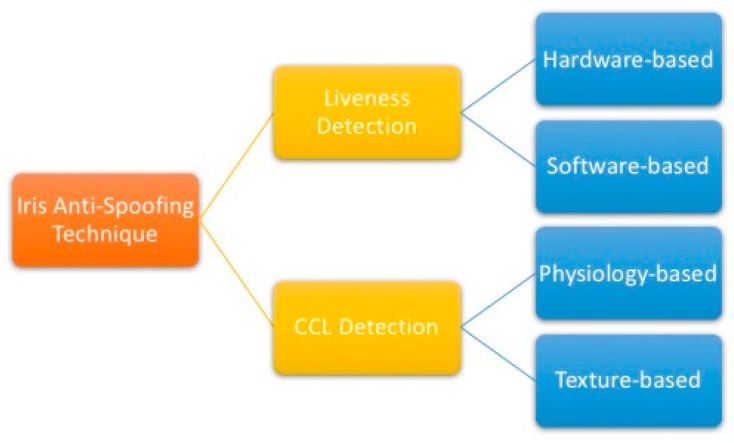
Summary of the division of the iris anti-spoofing technique.

To sum up, iris anti-spoofing techniques can be roughly divided into liveness detection and CCL detection, both with sub-categories. Figure 2 shows these research divisions. 

The literature survey in Section 2.1 and Section 2.2 shows that there was no universal solution to counter all counterfeiting attempts. Physiology-based detection is likely to handle most, but requires specific equipment additional such as illumination (spectral), modulation (temporal), sensors (spatial, spectral), and even more. On the other hand, a software solution with a robust classifier is relatively convenient yet it is uncertain whether the classifier is applicable for new types of CCLs [44,45,46]. Similar conclusions have been found in the field of fingerprint biometrics as well, as shown in [26,27,28,29].

In this study, we developed an iris imagery setup, which has capability to identify the subject without the need of self-cooperation. Taking advantage of high entropy density from the natural iris texture, we deduct the CCL occluded region from the natural iris pattern and leave the remainder for the recognition. To distinguish the CCL pattern and natural iris texture, we exploit their spectral distinctness and statistical independence in intensity, where the system involves two different spectral channels to capture the mixed iris images with different CCL-to-iris reflectance ratios. 

We consider our approach as an extremely novel one, since it combines the advantage of additional hardware (dual band camera system) and software (source separation algorithm). It does not rely on physiological or textural feature detection. Moreover, its major goal is for “recognition”, not just CCL “detection”. The final goal of the system is to perform iris recognition and achieve satisfactory results even under the condition that the subjects are wearing CCL. Therefore, our method does not belong to any sub-division of the existing method. The proposed method is a result from interdisciplinary research and should belong to a new category.

## 3. Framework of Dual Band Camera System

This section reveals the mathematical framework of the proposed dual band camera (DBC) system, which can be described by a 2 × 2 matrix. Based on the assumption that the natural iris texture and CCL pattern are statistically independent in spectral imaging formation (i.e., mixing matrix is full rank), we employed the independent component analysis (ICA) to estimate individual independent component (IC) of the mixed image, corresponding to either natural iris or CCL pattern. Compared to the natural iris, most CCLs have sharper histogram distribution due to its artificial pigments. We can easily determine the CCL by checking the individual histogram distribution. We thus designed a binary mask to remove CCL pattern from the mixed image, resulting in a CCL-free iris image for recognition process afterward. 

### 3.1. DBC Mixing Model

**Figure 3:**
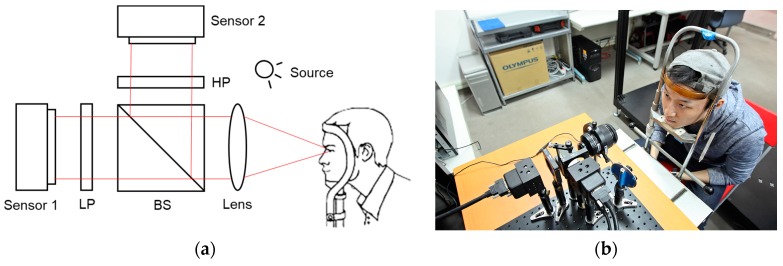
(**a**) The optical layout, where BS: beam splitter, LP: low-pass filter, HP: high-pass filter, the CCL-wearing subject was illuminated by a wideband white light source, two spectral channels (sensor 1 and sensor 2) thus retrieved different spectral imaging $y_{i}$ respectively; (**b**) real system implementation.

The imaging system is composed of a lens set and two sensors with distinct spectral filters (i.e., visible band-pass and NIR band-pass), as shown in Figure 3. Accompanying a wideband white illumination, the mixed image in each channel $y_{i}$(*x*) is captured with different albedo ($\rho_{ij}$) of natural iris texture ($s_{iris}\left(x\right)$) and the CCL pattern ($s_{CCL}\left(x\right)$), where *x* is the position index. Each channel $y_{i}$(*x*) is given by:

(1) $$\begin{array}{c} y_{1}\left(x\right)=\rho_{11}\cdot s_{iris}\left(x\right)+\rho_{12}\cdot s_{CCL}\left(x\right)\\ y_{2}\left(x\right)=\rho_{21}\cdot s_{iris}\left(x\right)+\rho_{22}\cdot s_{CCL}\left(x\right) \end{array},$$

$y_{1}$ and $y_{2}$ are mixed images captured by sensor 1 (visible) and sensor 2 (NIR), respectively. For notation simplicity, we omit the position index (*x*) in the following context.

### 3.2. ICA Algorithm

In general, it is convenient to represent Equation (1) in the vector-matrix notation as:

(2) y=As,

where $y={\left[\begin{array}{cc} y_{1} & y_{2} \end{array}\right]}^{T}$ is the mixed image set; $A=\left[\rho_{11}\;\rho_{12};\rho_{21}\;\rho_{22}\right]$ is the mixing matrix, and $s={\left[\begin{array}{cc} s_{iris} & s_{CCL} \end{array}\right]}^{T}$ is the source image set. The problem now becomes a classic blind source separation problem [47]. We aim to estimate the unknown mixing matrix A and look for its inverse, says W, to retrieve its original independent component $\hat{s}$:

(3) $$\hat{s}=Wy,$$

where W is called the demixing matrix. Since the demixing matrix W was estimated only using information contained in the mixed signal y, such an ill-posed problem cannot be solved without any prior information. As a result, an assumption was made that the natural iris texture $s_{iris}$ and CCL pattern $s_{CCL}$ have statistically independency. This naive assumption about independence is fairly legitimate since there is no reason to expect a physical correlation between the image of natural iris texture and artificial CCL pattern. Further examination will be made in later section. Given the linearity and uniformity of the image formation model, where both iris and CCL have a property of Lambertian reflectance, we can employ the ICA algorithm to separate two mixed images back to its original. 

In order to reduce the number of parameters to be estimated, a typical image pre-processing is necessary. Firstly, the data set y was centralized, i.e., the mixed images have zero-mean. Then we transformed the data set y into a new basis so that the components of new data set $\tilde{y}$ are uncorrelated with unity variances, so called whitening:

(4) $$\tilde{y}=\Lambda^{-1/2}Q^{T}y,$$

where Q and Λ are the eigenvector matrix and eigenvalue matrix of the covariance matrix $yy^{T}$. The advantage of the whitening process lies in that the new mixing matrix $\tilde{A}$ becomes orthogonal:

(5) $$\tilde{y}=\Lambda^{-1/2}Q^{T}As=\tilde{A}s.$$

As a consequence, the demixing matrix W for the whitened mixed image set ($\tilde{y}$) becomes orthogonal as well. The complexity in estimation of the demixing matrix W can be reduced from $n^{2}$ to n(n−1)/2 degree of freedom; n=2 in our case, the complexity was reduced from 4 to 1:

(6) $$\left[\begin{array}{c} {\hat{s}}_{iris}\\ {\hat{s}}_{CCL} \end{array}\right]\equiv \hat{s}=W^{T}\tilde{y}.$$

ICA is a simple and effective method for separating two independent signals. This technique begins with a pair of images taken through different spectral imagery channels. According to the central limit theorem, the mixed images with multiple independent sources tend to have a Gaussian distribution. On the other hand, the source image set, s, are relatively constrained to rarely Gaussian. We can pursuit the basis of W to maximize the non-Gaussianity of $W^{T}\tilde{y}$ and thus find the source image set $\hat{s}$ iteratively. Here, we used a standard fixed point algorithm and negentropy (i.e., a statistical measure of distance to Gaussian distribution) as the non-Gaussianity measure for the mixed image set. Due to the independency of source images, we can estimate ${\hat{s}}_{iris}$ and ${\hat{s}}_{CCL}$ by maximizing its negentropy:

(7) $$J\left(\hat{s}\right)=H\left({\tilde{y}}_{Gauss}\right)-H\left(\tilde{y}\right)$$

where J is the negentropy, H is the entropy operator, ${\tilde{y}}_{Gauss}$ is a Gaussian distributed random vector that has the same covariance matrix as $\tilde{y}$ does. According to information theory, a Gaussian distribution has the largest entropy for a given variance. Therefore, Negentropy is always nonnegative. To maximize the negentropy of $\hat{s}$, we used the gradient descent method subject to the condition ∥W‖ = 1. For more details about the ICA theory in this section, interested readers can refer to [47]. While the maximum negentropy was approached, the new basis of demixing matrix W can project the whitened mixed images $\tilde{y}$ back to its most likely independent origin $\hat{s}$, as the estimated source image set involving CCL and natural iris texture. 

### 3.3. Restrictions and Ambiguities in ICA

At this point, the validity of ICA in the context of this work should be investigated. Firstly, we assumed the surface properties of the CCLs and natural irises to be Lambertian. That means the intensity at each point in the mixed image was a linear superposition from the source images $x_{iris}$ and $x_{CCL}$ homogeneously. This assumption doesn’t fully stand but rational due to the reason that the reflective scattering distribution from both random textures is fairly similar. As a consequence, the Lambertian model was fairly close to the ground truth. Secondly, two source images $x_{iris}$ and $x_{CCL}$ were assumed to have statistical independence. This assumption holds because there is no evidence showing that there exists a strong correlation between the natural iris texture and the CCL pattern in manufacturing. Thirdly, the histograms of individual source images must have non-Gaussian distributions. Since the CCLs were patterned by a specific ink material, the histogram of CCL appears a narrow distribution, far different from the Gaussian shape. But for the natural iris whose texture was attributed by richer pigment compositions, the histogram involves more grey levels and was relatively broad. In this study, both histograms were non-Gaussian after measurement. At last, the number of mixed images and source images should be equal, thereby avoid the problem to be underdetermined. This concern is closely associated with the physics of spectral mixing. To account for the case that the mixing matrix is non-square, we could employ a more generic version of ICA that allows for degenerate (or redundant) spectral channels. But for the purpose of proof of concept, we reinforced the independency through the spectral design of two distinct filters in this study.

Numerous studies have disclosed that ICA suffers from two inherent ambiguities in the recovery of source image sets. The first one lies in the scaling ambiguity, where we cannot determine the variances of the estimated source images $\hat{s}$. The mixing model in Equation (5) can be rewritten as:

(8) $$\tilde{y}=\sum_{i}\left(\frac{1}{\alpha_{i}}\rho_{i}\right)\left(\alpha_{i}s_{i}\right),$$

where the scalar multiplier $\alpha_{i}$ in one of the source image patterns is cancelled out through division by the corresponding column $\rho_{i}$ of A. Each factor in the element of $\alpha_{i}s_{i}$ represents identical image patterns, but each is scaled by $\alpha_{i}$. This indicates that the energy (histogram) of the image is shifted. The scaling ambiguity would lead the estimated intensity of source images different with the ground truth. In fact, for the iris recognition, we always employ post histogram specification to enhance the image contrast and counter the brightness inconstancy from the illumination. Fortunately, the intensity disparity doesn’t affect the accuracy for the purpose of iris recognition.

The second ambiguity is the order ambiguity, where it is unlikely to determine the order of the source images $\hat{s}$. To show this point, Equation (5) can be rewritten as Equation (9):

(9) $$\tilde{y}=\left(\tilde{A}P^{-1}\right)\left(Ps\right),$$

where P is the permutation matrix. The factors in the element of Ps represent the same image pattern set, but in a different order. This indicates that the order of two estimated source images (${\hat{x}}_{iris}$, ${\hat{x}}_{CCL}$) is ambiguous. This issue can be overcome by analyzing individual histogram, detailed procedure will be disclosed in Section 4.2.

## 4. Design of Dual Band Camera System

### 4.1. Filter Design via Spectral Response

**Figure 4:**
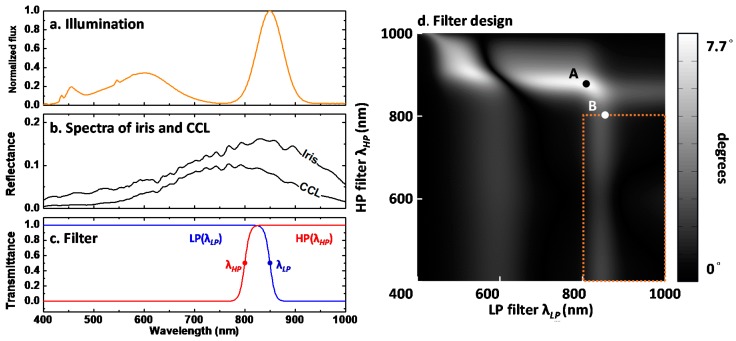
(**a**) The spectra of illumination; (**b**) the albedo of iris and CCL; and (**c**) the transmittance of two designed filters. The $\lambda_{LP}$ and $\lambda_{HP}$, cutoff wavelength of each filter, were used as the design parameter. (**d**) The span angle space with respect to cutoff wavelength pairs. Orange rectangle represents the energy constrain. Although point A ($\lambda_{LP}=808\;\mathrm{nm}$, $\lambda_{HP}=879\;\mathrm{nm}$) has largest span angle between two spectral components, but no crossed transmission window led to string energy loss. As a result, the point B ($\lambda_{LP}=850\;\mathrm{nm}$, $\lambda_{HP}=800\;\mathrm{nm}$) was selected as the operation point, allowing a transmission window around 800–850 nm in NIR.

The dual band camera (DBC) system was equipped by two distinct spectral filters to create full rank conditions. In addition to well-conditioned, we have to ensure both channels have a sufficient amount of energy. The optimization of the mixing matrix was crucial for the success of ICA. To comply with the matrix-vector notation y=As for the spectral analysis, we split the source image into two terms: one is the object pattern, s, which is wavelength independent. The other is the corresponding albedo, $R_{i}\left(\lambda \right)$, which is a function of wavelength as well as other experimental parameters such as source luminance L(λ), sensitivity of the sensor S(λ) and the transmission of the filter $F_{j}\left(\lambda \right)$, respectively. The wavelength dependent terms integrated over the entire spectrum would be lumped into the entries ($\rho_{ij}$) of the mixing matrix $A=\left[\rho_{11}\;\rho_{12};\rho_{21}\;\rho_{22}\right]$ as:

(10) $$\rho_{ij}=\;\sum_{\lambda =400}^{1000}F_{j}\left(\lambda \right)\cdot S\left(\lambda \right)\cdot L\left(\lambda \right)\cdot R_{i}\left(\lambda \right),$$

where index i corresponds to an object and j represents the selective filter. Note that except for the object pattern s, all the parameters are dependent on the wavelength. The mixed image response was attained by integration over the entire spectral response of all the devices. In this study, we maximize the separation between two mixed images s via optimization of the mixing matrix A via filter design $F_{j}\left(\lambda \right)$ only, whereas all other wavelength-dependent terms were kept equal in both channels. Figure 4a,b shows the normalized luminance and statistical average over the tested subjects in this work. We can observe that the albedo of the (East Asian) subject’s iris and CCL under test are very similar, led to a challenge for ICA process. We employed two distinct filters characterized by the cutoff wavelength, as shown in Figure 4c. Here we denoted $y_{1}$ as the first mixed image with low-pass filter (LP filter, cutoff wavelength $\lambda_{LP}$) and $y_{2}$ as the second mixed image with high-pass filter (HP filter, cutoff wavelength $\lambda_{HP}$). The mixing matrix A is well-conditioned (full rank). Since the purpose is to make mixed image set y having the possibly largest independency for ICA, we aimed to find the adequate basis of the mixing matrix A through the filter design. The span angle of basis for row space of A was calculated through different cutoff wavelength pairs ($\lambda_{LP}$, $\lambda_{HP}$). Here we adopted $\det\left(A\right)/∥A_{2}\|$ as the merit to evaluate the span angle, where the determinant of A describes the independency and the *L*2 norm of A represents how much energy it holds.

As shown in Figure 4d, the maximal angle occurred at point A ($\lambda_{LP}=808\;\mathrm{nm}$, $\lambda_{HP}=879\;\mathrm{nm}$) with 7.7° in global search. However, no crossed transmission window would result in strong energy loss, thus degrades the signal to noise ratio of the captured image. As a result, we set a regulation that the total transmission energy should be over 50%. The energy regulation, attributed by the *L*2 norm of A, was expressed as the right bottom rectangle of Figure 4d. To tradeoff the maximal independency and energy constrain, we selected the operational condition ($\lambda_{LP}=850\;\mathrm{nm}$, $\lambda_{HP}=800\;\mathrm{nm}$) with span angle 2.8°, as shown in the point B.

**Figure 5:**
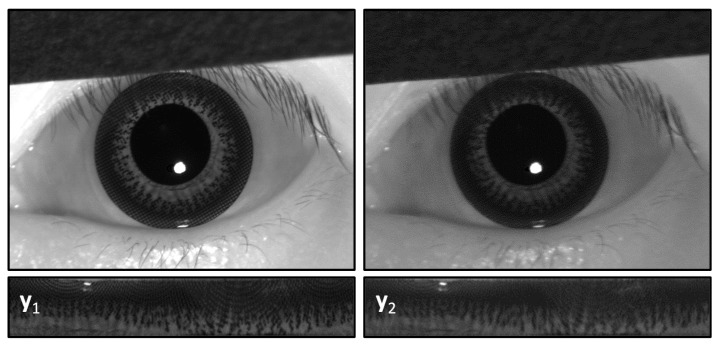
Iris images captured through the LP filter ($y_{1}$) and HP filter ($y_{2}$), respectively. At the current step, both mixed images are the combination of two source image, $x_{iris}$ and $x_{CCL}$, with different ratio. The upper row is in Cartesian domain; and the lower row is in polar domain.

The captured iris images ($y_{1}$, $y_{2}$) are revealed in upper row of Figure 5, respectively. A series of image processing steps was conducted for iris recognition. Firstly, the iris images were segmented by determining the centers and radii of the pupillary and limbic boundaries. Then the iris images were normalized by transforming the coordinates from Cartesian to polar, as shown in the lower row. 

### 4.2. Mask Generation

**Figure 6:**
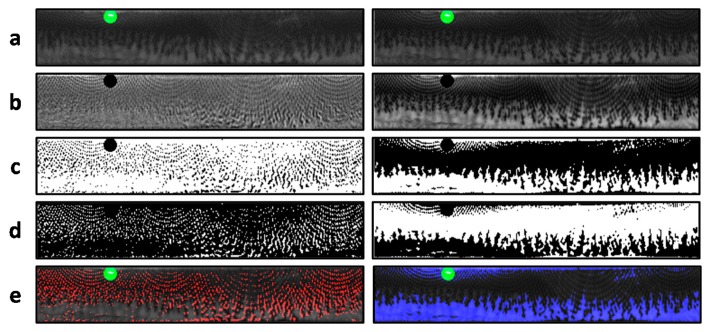
(**a**) The normalized images with specular points being removed; (**b**) Two estimated ICs through the ICA; (**c**) the binary masking of the ICs; (**d**) the complementary masks from the ICs; and (**e**) the inference two masks on the mixed image. The red and blue marks denote the inference of CCL pattern and iris texture, respectively. The green mark denotes the specular mask. It is noted that red mask had higher negentropy (as the Non-Gaussianity measure) in histogram analysis, thus esteemed as the CCL pattern.

Before conducting ICA to separate the textures on the basis of two estimated components, we must remove the specular points which do not satisfy the linearity mixing and lead to errors. We used a simple mask to remove the specular points shown in green mark of Figure 6a. Then, ICA algorithm in Section 3 was applied to estimate two ICs ($\hat{s}$), as shown in Figure 6b. 

As mentioned in Section 3.3, the ICA algorithm has two ambiguities to tackle. The first ambiguity is the scale uncertainty. The scale uncertainty includes the energy uncertainty (the range of the grayscale could be any value) and sign convention uncertainty (either positive or negative value can be true texture), leading to the challenge in threshold. The second ambiguity is the order of two estimated solutions. We cannot confirm either to be iris or CCL pattern. To ease the problem, the ambiguity issue can be solved by finding the CCL pattern only. As long as CCL pattern was determined, the natural iris texture can be attained accordingly.

### 4.3. Overcoming the Scaling Ambiguity

Generally, the pixel values of two estimated source images are continuous. To discretize the CCL texture in template comparison, we used binary thresholding to solve the problem of the energy uncertainty. The binary thresholding transforms the greyscale patterns from the image into a binary mask. Ideally, the estimated pigment pattern would have full grayscale value, and zero for otherwise. In this work the threshold level was set at 0.5 to extract the pigment pattern accordingly. Figure 6c shows two binary masks, where the white region presented the estimated components whose greyscale was higher than threshold value 0.5. In the following we created two complementary masks to tackle the problem of sign convention uncertainty, as shown in Figure 6d. The white region represents the estimated components whose greyscale was lower than threshold value 0.5. The distinct clues of the CCL and iris texture was easily appeared through the binary mask.

### 4.4. Overcoming the Order Ambiguity

To further inspect the distinctness between natural iris and CCL, the negentropy (as the non-Gaussianity measure) was used again. The albedo of CCLs is contributed to by the artificial pigments, whose histogram in spectral image tends to have a sharply peaked distribution. In contrast, the histogram of natural iris images appeared more likely Gaussian whereas the albedo widely varied among the iridal region with different ratio of natural pigment components (such as eumelanin and pheomelanin). Therefore, we used the negentropy to distinguish the order of estimated source images between CCL and natural images. The features after masking appeared more observable, as shown in the Figure 6e. We colored the estimated CCL and iris regions by red and blue marks, respectively.

**Figure 7:**
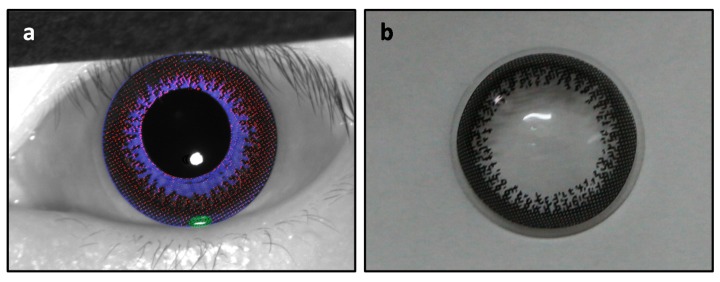
(**a**) The estimated CCL (colored in red) and remaining natural iris pattern (colored in blue) were visualized for the comparison purpose, where the annular dot pattern at the outer boundary were in close agreement with (**b**) ground truth CCL picture.

Figure 7a colored two masks in Cartesian coordinate for feature visualization. The mask in red displayed an annular dot pattern at the outer boundary around the iridal region. The texture of this estimated component (esteemed CCL) were in close agreement with the ground truth CCL picture in Figure 7b. The mask in blue displayed a sunflower shape, which was the remaining part of the iris texture. 

## 5. Proof-of-Concept Testing

### 5.1. Setup and Database

The camera system was equipped with a NIKKOR-W lens (Nikon, Tokyo, Japan) with focal length 210 mm and F-number 5.6. A one-inch 50/50 non-polarizing beam splitter (BS014, Thorlabs, Austin, TX, USA) splits the optical path into two branches. Two designed spectral filters were placed of the two sides of the beam splitter, where the LP filter with 850-nm cutoff wavelength (#64-670 EO Optics, Barrington, NJ, USA) and HP filter with 800-nm cutoff wavelength (EO Optics #64-705). The optical system came with two CMOS image sensors (MV1-D2080-160-CL-12, Photonfocus, Lachen, Switzerland), acquired images up to a resolution of 2080 × 2080. The working distance was set to 2 m.

We collected iridal data from ten East Asian people as the proof-of-concept dataset. Each person has two irises; therefore, there were totally 20 iris classes. For each class, ten images were captured for enrollment, and another ten image pairs were captured in both spectral channels. A total of 200 enrollment images and 200 test image pairs were acquired. The subjects wore a type of contact lenses from TICON, where the CCL pattern was formed by a circular band comprising random dots.

### 5.2. Iris Recognition Algorithm

Iris recognition algorithm has been developed for more than a decade. In this work, we mainly adopt the iris recognition algorithm proposed by Daugman, which is one of the earliest and most cited works in this field [1]. Generally, the whole iris recognition process consists of the following stages: iris image acquisition, iris segmentation, iris normalization, feature extraction and feature matching. The iris image acquisition process is to capture iris images using optimized optical devices. The iris segmentation process finds the inner and outer boundaries of the iris region. The normalization process transformed the iris image from Cartesian coordinates to polar coordinates. Iris image normalization can prevent performance degradation caused by the tilting of the subject’s head or off-axis gazing. For feature extraction, in our approach, a one-dimensional Log-Gabor filter was applied to extract prominent features as an iris code. The features were thus quantized into binary codes according to their quadrant information on the complex plane.

Two iridal codes can be matched using a bit-wise XOR operation, which obtains the Hamming Distance (HD) between the two irises. Small HD indicates that the two irises were more likely belong to the same class (i.e., it is a case of authentic comparison). On the other hand, higher HD indicated a statistically independent relationship between the two irises, implying the two irises belong to different classes (i.e., it is a case of impostor comparison). An appropriate threshold value of HD was set to determine the acceptance or rejection of the iris image. In our experiments, four quantitative merits were used to examine the system performance, including: false acceptance rate (FAR), false rejection rate (FRR), equal error rate (EER) and sensitivity index (SI), respectively.

### 5.3. Baseline Testing

**Figure 8:**
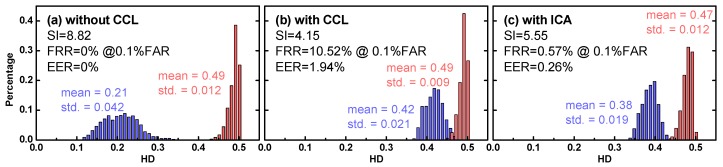
Experimental HD histogram for the authentic (blue) and impostor (red) distribution with 200 enrollments and 200 test images as the proof of concept, where (**a**) naked eye (**b**) CCLs and (**c**) CCLs with the ICA, respectively.

With a total of 200 enrollment images and 200 test naked eye image as the baseline test, the recognition system had performance as SI = 8.82, FRR = 0% when FAR was set to 0.1%, and the EER was 0%, as shown in Figure 8a. For a recognition system with high accuracy, the two distributions should have a minimal or no overlapping region. The results indicated that our system was as accurate and robust as those presented in the Iris Challenge Evaluation (ICE) 2006, an iris recognition grand challenge that employed a data set comprising 29,056 right iris and 30,502 left iris images; the three highest-performing systems in the ICE 2006 had FRR ranges of roughly 1–2.5% when the FAR was set to 0.1%, according to [48].

When the subjects wore CCLs, both FRR (10.52%) and EER (1.94%) increased dramatically in comparison with the naked eye, as shown in Figure 8b. Since the CCL patterns in the test have no correlation with natural iris texture, the authentic distribution was shifted rightward and overlapped with the retained imposter distribution, thus deteriorating the recognition accuracy. Such results were used as the baseline to examine the effect of proposed ICA algorithm.

### 5.4. Results of ICA-Based Recognition against CCL Spoofing

After conducting ICA and masking process for CCL-wearing subjects, the authentic and impostor HD distribution was separated farther (Figure 8c), with moderate FRR (0.57%) and EER (0.26%). Compared with the baseline (without ICA), yielding an 18.5× reduction in terms of FRR. The leftward shifting authentic distribution manifested the success of proposed ICA algorithm that the CCL pattern was mostly removed and discounted in the iris recognition. No surprise that it is unlikely to have the comparable performance with the naked eye, because the CCL masking would reduce the entropy density in natural iris. Additional noise arose from PCA also contaminated the ground truth data in a small amount. Certainly, when the spectral distinctness between the CCL and natural iris increases, the ICA-based scheme to separate the CCL and natural iris texture was expected to be more effective.

## 6. Conclusions

In this work, a dual-band camera system was proposed to tackle CCL-based spoofing attempts in iris recognition systems. Based on the statistical independency and spectral distinctness between the natural iris texture and CCL, we successfully removed CCL patterns from the natural iris textures by applying the ICA algorithm with high-order statistical moments estimation. The concept was preliminarily proved that the FRR was significantly reduced down to 18.5× compared with an ICA-free recognition system.

The novelty of proposed scheme is that it does not require prior knowledge about various types of CCL, thus has potential to tackle new types of CCL. At its current status, the mixing matrix was constrained to be well-conditioned, which corresponds to the equal dimension between the source image (CCL and iris) and mixed image (two spectral channels). As long as the mixing matrix is full rank, the separation of natural iris and CCL can be effectively separated through the ICA algorithm. For our application at conceptual level, we restricted ourselves to the case of just two mixed images since the experiment was mainly focused on the single color type CCLs. When people wore CCLs that have more than one color, the multiple ICs with specific color would lead to maximal negentropy via some linear combination of the primitives. Insufficient channels with ICA for multiple color type CCL are worthy of further investigation, as is spectral analysis differences between irises of different races’ and CCLs with artificial pigments. Given the simplicity of calculation and versatility of the proposed setup, our scenario is expected to be helpful for resolving underlying spoofing issues.
